# Study of the Accumulation of Toxic and Essential Ultra-Trace Elements in Fruits of *Sorbus domestica* L.

**DOI:** 10.3390/ijerph14040341

**Published:** 2017-03-24

**Authors:** Michaela Zeiner, Iva Juranović Cindrić, Boris Majić, Gerhard Stingeder

**Affiliations:** 1 Division of Analytical Chemistry, Department of Chemistry, University of Natural Resources and Life Sciences, BOKU, Muthgasse 18, 1190 Vienna, Austria; gerhard.stingeder@boku.ac.at; 2 Department of Chemistry, Faculty of Science, University of Zagreb, Horvatovac 102a, Zagreb 10000, Croatia; ijuranovic@chem.pmf.hr (I.J.C.); boris.tybs@gmail.com (B.M.); 3 Department of Biology, Faculty of Science, University of Zagreb, Horvatovac 102a, Zagreb 10000, Croatia

**Keywords:** *Sorbus domestica* L., ultra–trace elements, ICP–SFMS, metal accumulation, climatic influence

## Abstract

In the present work, the accumulation of selected toxic and essential ultra-trace elements in fruits of service tree (*Sorbus domestica* L.) were determined depending on harvest time. Samples were collected from the same sampling area in two different years and within one year in September and October (maturity state). Harvesting the fruits in the same area excludes the influence of metals taken up via roots, thus the impact of airborne contamination by heavy metal translocation can be studied. All samples were dried and digested using an acidic microwave assisted digestion system prior to quantification by inductively coupled plasma—sector field mass spectrometry (ICP–SFMS). The elements chosen were Arsenic and Cadmium as well as Lithium, Molybdenum, and Selenium. The Arsenic content rose with maturity in mesocarp. Cadmium found in the mesocarp was unaffected by ripeness. For Selenium and Molybdenum, no statistically significant effect of ripeness could be found on their content in mesocarp. Lithium could not be detected in the majority of fruit samples. Differences between the metal concentrations based on the year of harvest were found for Arsenic, Molybdenum, and Selenium, depending on precipitation. The drier the season, the more Arsenic was accumulated. For Molybdenum and Selenium, the opposite effect was observed.

## 1. Introduction

The service tree (*Sorbus domestica* L.), a deciduous species belonging to the *Rosaceae* family, has been used for nutritional and construction purposes since Roman times. It is distributed in southern and central Europe, northern Africa, Asia Minor and Crimea [1]. Whereas the fruits have been served as food, the dense and highly valuable tough wood has been used for preparing various mechanical parts. Today, its main uses are as skeletal tree for windbreaks and refuges for wildlife [2], apart from nutritional purposes [3]. The consumption of the fruits is correlated with the control of diabetic complications [4]. Furthermore, in Turkey (Kırklareli Province), this plant is considered as one of the twenty most used traditional medicinal plants [5]. The fruits are directly consumed and also as processed products such as jam, juice and brandy. The anti-oxidative activity is one of the main reasons for the nutritional importance. Ölschläger and colleagues [6] described the phenolic content of the service tree fruits including procyanidins, cinamic acids and flavonoid quercetin. The correlation between maturity state and anti-oxidative activity was investigated by Termentzi et al. [7], whereby this property decreases with ripeness. This change is not based only on quantitative differences, but also on qualitative ones [3]. Furthermore, the phenolic content was found to strongly dependent on the species of *Sorbus* L. fruits [8]. Water extracts of wild service tree fruits (*S. torminalis* L.) has been found to be an important natural source of acetylcholinesterase inhibitors [9]. Not only the fruits of *S. domestica* L. are good contributors to nutrition, but also aqueous bark extract is used for stomachache and ulcer treatment [5], but without data on its phenolic content. Furthermore, the content of tannins (phenolics with defensive role) in different parts of service tree is still not revealed.

Apart from phenolic compounds, other components and fruit parameters, such as skin and flesh firmness, color or flavor, change with fruit growth and ripeness [10]. Therefore, the mineral composition in the fruit is supposed to also change with rising maturity. In spring, essential elements are more accumulated in leaves than in autumn, when harmful elements are found in higher concentrations, since, by losing leaves in fall, the plant can get rid of toxic metals. Furthermore, the nutritional value of a plant and its extracts is also attributed to its inorganic compounds (minerals) [11]. Currently there are few scientific papers on the mineral element composition of service tree species [12].

According to WHO, Arsenic has been associated to various health problems, especially in cases of early-life exposure [13,14]. Main uptake routes of this element are drinking water and food. Acute symptoms are vomiting, abdominal pain and diarrhea, and long time exposure leads to skin problems including cancer, as well as cancer of lungs and bladder [15].

Cadmium is another harmful element, wide spread in the environment. It is easily taken up and accumulated by plants and crops through the roots. The resulting health effects are strongly correlated with the route of exposure. The main target organs of cadmium are the lungs, kidneys and the bones [16].

Lithium can reduce symptoms of different types of depression and anxiety [17]. It occurs as natural trace element and is mobilized by rain from rock and soil, thus it may enter water and can be taken up by plants. Thus, human dietary lithium intakes depend on location and the types of foods consumed, and vary over a wide range [18].

Molybdenum is considered to be an essential element, being required for different enzyme systems, such as for making red blood cells. Molybdenum deficiency may lead to neuropsychiatric disorders [19].

Selenium is also an essential element for humans and animals. It is attributed to protection against heavy metal toxicity [20]. The concentration range in which Selenium is essential is narrow, low concentrations can cause anomalies in organisms whilst high concentrations are toxic. Contents in food ranging from 2 to 8 mg/kg are considered to be harmful [21].

The aim of the present study was the determination of As, Cd, Li, Mo, Pb, and Se in service tree fruits (mesocarp) of different ripeness states collected in Croatia. Furthermore the influence of the year of harvest on the elemental concentrations was investigated. The metals taken up from the soil are supposed to be in a similar range when harvesting the fruits in the same area, thus the impact of airborne contamination by heavy metal translocation can be studied.

## 2. Materials and Methods

### 2.1. Samples

Service tree (*Sorbus domestica* L.) fruits were collected in Mađari (Croatia; 45°29′ N 16°22′ E) in September 2009 and 2011. The fruits were of two or three different ripeness states: immature fruits (yellow color), ripe fruits (brown color) and well-matured fruits (windfall—only 2011). For each subcategory five samples were taken. The fruits were frozen under liquid nitrogen, lyophilized (Christ Alpha 1–2, Christ, Osterode am Harz, Germany, −60 °C, 0.01 mbar, 24 h) and separated in exocarp, mesocarp and seeds. Mesocarp was used for further investigation. Before analysis, the samples were homogenized in a metal-free mortar.

### 2.2. Chemicals and Reagents

Nitric acid (HNO_3_) and hydrogen peroxide (H_2_O_2_) were purchased from Sigma (Munich, Germany) in suprapure quality for trace analysis. The multielement standard (ICP Multielement Standard IV) was purchased from Merck (Munich, Germany). The standard reference material of strawberry leaves (LGC7162) was obtained from LGC Standards (London, UK). Ultra-pure water was prepared in an in-house instrument.

### 2.3. Sample Preparation

MWS–2 Microwave System Speedwave Berghof was used for the microwave assisted digestion of the fruit samples. This digestion system has an output power of 1000 W. Ten samples can be digested in one cycle. In each round, nine samples and one blank were run. Approximately 100 mg weighed to the nearest 0.1 mg of the lyophilized sample was put into a Teflon reaction vessel (in duplicate). Then, 5 mL HNO_3_ (50:50 *v*/*v*) and 1 mL H_2_O_2_ (1 mol/L) were added. The digestion consisted of three 15 min steps (1. −*T* = 110 °C, 2. −*T* = 170 °C and 3. −*T* = 140 °C). The resulting clear solutions were made up to 10.0 mL with ultra-pure water.

### 2.4. Measurements

The elements were quantified with ICP–SFMS (Element 2 ICP–SFMS from Thermo Fisher, Bremen, Germany). The instrument was equipped with a self aspirating PFA microflow nebulizer (ESI; flow of 100 µL/min), a PC^3^ cyclonic quartz chamber (ESI; operated at 4 °C), a quartz injector pipe and torch, aluminum sampler and skimmer cone (all from Thermo Fisher). The instrumental conditions applied were: RF power, 1300 W; plasma gas flow, 16 L/min; sample gas, 1.06 L/min; and auxiliary gas flow, 0.86 L/min.

The isotope ions were analyzed at different resolutions, low resolution (^7^Li^+^, ^82^Se^+^, ^111^Cd^+^, and ^208^Pb^+^), medium resolution (^98^Mo^+^) and high resolution (^75^As^+^), the nominal mass resolutions being 350, 4500 and 10,000, respectively. At all resolution levels, Indium (^115^In^+^; 1.1 μg/L) was used as internal standard.

### 2.5. Optimization and Characterization of the Analytical Method

The trueness of the method for the fruit digests was estimated by analyzing strawberry leaves reference material five times. The overall repeatability of the instruments was determined by analyzing selected samples (digests of unripe and ripe fruits collected in 2011; n = 2 + 2) after calibration on two different days. The precision given as relative standard deviation (RSD) was evaluated by measuring certain samples five times. The sensitivity of the methods was evaluated for each metal using the obtained slope of the calibration curve. The limits of detection (LOD; 3 *σ*) and limits of quantification (LOQ; 10 *σ*) were calculated according to Boumans [22].

### 2.6. Statistical Analysis 

All analyses were carried out in three replicates and the obtained data were presented as means ± SD (standard deviation). Statistical significance of the differences between the investigated groups was evaluated by paired *t*-test, based on *p* < 0.05.

## 3. Results and Discussion

### 3.1. Analytical Method

The obtained validation data for the analytical method used are in the range for the determination of trace elements in biological samples. The LODs and LOQs for the digested flowers and leaves are listed in Table 1.

The recoveries determined with the standard reference material of strawberry leaves for As, Cd, Mo, and Pb are presented in Table 2. For each analyte, the range (i.e., minimum and maximum value) and mean of the obtained value are given besides the mean recovery. For Li and Se, only indicative values are given for the standard reference material of strawberry leaves due to inhomogeneity of the material. The indicative contents are 0.7 mg/kg and 0.04 mg/kg, respectively. In the presented study, the following data were obtained, given as mean/minimum/maximum (all in mg/kg) 0.623/0.594/0.652 for Li and 0.0347/0.0297/0.0398 for Mo. The calculated mean recoveries for these elements are 89% and 87%, respectively.

The sensitivity of the method was evaluated by the slopes of the calibration curves for the analytes, all coefficients of determination (R²) being higher than 0.9990. The precision obtained was in the range from 0.5% up to 2.8% and the day-to-day repeatability from 1.2% to 2.6%. The overall uncertainty of measurement lies in the range from 2.6% to 4.8%.

### 3.2. Metal Content in Fruits

Samples of service tree fruits (mesocarp) were analyzed for selected ultra-trace elements after microwave digestion in aqueous 50% HNO_3_ (*v*/*v)*. The obtained results for all analytes expect for Li are listed in Table 3. Lithium was found only in the ripe fruits from 2009, with a maximum value of 0.063 mg/kg and a median of 0.047 mg/kg. Since one value was below LOD, the calculation of a mean value is not useful. In all other samples Lithium contents were between LOD and LOQ.

Currently, there are no data on these elements in service tree fruits published in scientific journals. One paper reports elemental concentrations in various parts of this plant, but only regarding minor and major elements as well as organic compounds [12]. Majic and colleagues do not distinguish between the grade of ripeness and the year of harvest. Thus, no comparison with literature data is possible. The present amounts of the toxic elements are in a range where they are not supposed to have negative impact on human health when consumed.

The quantitative determination of minerals in food stuff, especially in fruits and berries is important, since their concentration can influence the quality of fruit, like organoleptic characteristics and stability, and after consumption the health status of humans. Regular consumption of traditional fruits, such as the investigated service tree fruit, is supposed to potentially improve national diet along with other health influencing factors [23]. The results obtained in present investigation were tested for statistically significant differences in dependency of the year of harvest (2009 and 2011) as well as between the different ripeness states (immature, ripe and windfall). Regarding the influence of the harvest year, it can be stated that the chemical composition and thus the nutritional value of fruits are dependent not only by the species/cultivar, but also by many other factors, such as production area, climate and soil, agricultural practices (organic or classical) and quality of the irrigation water. After harvest, the way and duration of storage and further processing may also change the chemical composition. From these parameters listed above, soil and agricultural practice are not of importance for the samples of this study, since they were naturally grown without anthropological influence and collected from the same area. Climate conditions are similar but the amount of rain and thus deposition of contaminants varies from year to year. For the sampling, the rain and temperature data are given in Table 4.

It can be seen from Table 5 that the beginning of the year 2011 was warmer and drier than 2009 from January till March. In addition, from June to September the amount of precipitation was lower. These facts can be compared with the contents of the determined elements in the fruits collected in these two years. Whereas the Cadmium content is unaffected by the climatic conditions, Arsenic and lead were found in higher amounts in the drier year 2011. On the other hand, Molybdenum and Selenium were present in the fruits in higher contents in 2009. Thus, it is plausible that these elements are the result of environmental pollution and wet deposition. Since Lithium was mainly found only in concentrations between LOD and LOQ, no conclusion can be drawn on the influence of harvest year.

The second objective of the present paper was to see the dependency of elemental content on ripeness. For organic compounds, it has been found that ripeness stage at harvest time affects fruit composition and thus their quality [10,28]. Regarding service tree fruits, unripe yellow fruits, together with the fruit pulp, were found to be the strongest antioxidants, while the well-matured brown fruits were the weakest ones [4]. Furthermore, significant qualitative and quantitative differences in the phenolic content depending on the maturity stages were observed [7]. Thus, it was of interest, whether the elements are also present in the fruits in different contents according to ripeness state. Since Lithium was mainly found only in concentrations between LOD and LOQ, no conclusion could be drawn regarding correlation between ripeness and elemental concentration. Cadmium shows a slight increase with ripeness, but this rise is not statistically significant. Arsenic shows a similar behavior to Cadmium. Its contents in the mesocarp are slightly rising, but without statistical significance. For Molybdenum, no effect of the ripeness state on its amount could be found, in 2009 and 2011. Regarding Selenium, it can be seen in Table 3 that the content decreases slightly, but not statistically significant with the ripening process in 2009. The same tendency can be concluded for 2011, when selenium was found only in the immature fruits. Due to results below LOD for ripe and windfall fruits in 2011, no statistical test could be performed to underlie this finding. For lead, a diverse pattern was registered for both years. Whilst in 2009 the content decreases from unripe to ripe state, in 2011 there is a rise in this period followed by a decrease only in the end towards the windfall state.

## 4. Conclusions

Based on the results discussed above, it can be concluded that service tree fruits can be considered as healthy food regarding their content of toxic elements. On the other hand, they contain non-negligible quantities of essential trace elements, such as Selenium and Molybdenum, but cannot be used as source of Lithium.

Regarding the influence of the year of harvest on the element contents, it can be seen that all of the ultra-trace elements studied show different accumulation behaviors. Correlation with climatic factors led to the conclusion that Arsenic is present in higher amounts in drier years, whilst Selenium and Molybdenum contents rise with precipitation. Cadmium content was unaffected by the year of harvest.

The ripeness state has only little influence on the contents of the elements investigated. None of the slight increases and decreases found are all statistically significant.

## Figures and Tables

**Table 1:** LOD and LOQ for all analytes in digest solution (µg/L) and fruit material (mg/kg).

Limit of Detection/Quanitfication	As	Cd	Li	Mo	Pb	Se
LOD (3 s) μg/L	0.003100	0.000330	0.099000	0.013000	0.001400	0.062000
LOQ (10 s) μg/L	0.010000	0.001100	0.330000	0.044000	0.004800	0.210000
LOD (3 s) mg/kg	0.000310	0.000033	0.009900	0.001300	0.000140	0.006200
LOQ (10 s) mg/kg	0.001000	0.000110	0.033000	0.004400	0.000480	0.021000

Note: LOD: limits of detection; LOQ: limits of quantification.

**Table 2:** Results for As, Cd, Mo, and Pb in CRM, certified values, and recoveries.

	As	Cd	Mo	Pb
Found (mg/kg) mean minimum–maximum	0.276 0.271–0.284	0.186 0.174–0.194	0.300 0.271–0.318	1.78 1.72–1.83
CRM (mg/kg)	0.28 ± 0.07 *	0.17 ± 0.04 *	0.32 ± 0.08 *	1.8 ± 0.4 *
Recovery (%)	98	110	94	99

* 95% confidence interval.

**Table 3:** Minimum–mean–maximum metal content in fruit material (mg/kg) *n* = 5.

Year of Harvest	Maturity State	As	Cd	Mo	Pb	Se
2009	immature	<LOD	0.010–**0.018**–0.021	0.94–**1.2**–1.4	0.082–**0.11**–0.13	0.28–**0.35**–0.37
	ripe	0.054–0.19 *–0.23	0.022–**0.024**–0.026	0.94–**1.3**–1.5	0.081–**0.089**–0.098	0.26–**0.28**–0.31
2011	immature	0.23–**0.40**–0.46	0.012–**0.019**–0.029	0.68–**0.76**–0.82	0.098–**0.14**–0.16	0.078–**0.088**–0.11
	ripe	0.47–**0.61**–0.84	0.010–**0.015**–0.022	0.76–**0.81**–0.83	0.12–**0.19**–0.26	<LOD
	windfall	0.46–**0.54**–0.58	0.013–**0.025**–0.030	0.74–**0.77**–0.81	0.14–**0.15**–0.17	<LOD

* Bold value = mean.

**Table 4:** Climate conditions in Sisak (HR) 2009 [24] and 2011 [25].

Month	2009		2011	
	Temperature *	Rain *	Temperature *	Rain *
January	40	96	83	9
February	67	43	39	7
March	72	46	67	3
April	>99	8	96	8
May	98	19	75	10
June	71	95	95	84
July	96	99	92	67
August	99	21	>99	25
September	92	10	99	10

* Expressed as percentage of norms 1961–1990.

**Table 5:** Detailed data for temperature and precipitation in Sisak (HR) in 2009 and 2011 [26].

Month	2009		2011	
	Temperature (°C) *	Rain (L/m^2^)	Temperature (°C) *	Rain (L/m^2^)
January	0.0	113.2	0.1	15.3
February	1.4	40.4	0.8	13.5
March	4.8	53.6	4.4	21.0
April	11.5	33.3	11.0	31.2
May	15.9	44.2	12.1	31.7
June	14.0	153.2	18.7	125
July	20.5	171.4	19.7	88.3
August	20.4	37.3	20.6	42.0
September	14.9	30.2	16.0	30.2

* Calculated from the percentage given in Table 4 and the mean monthly temperature reported for Sisak [27].
